# The Clinical Phenotype of Binge Eating Disorder among Postmenopausal Women: A Pilot Study

**DOI:** 10.3390/nu15092087

**Published:** 2023-04-26

**Authors:** Savannah C. Hooper, Sara E. Espinoza, Victoria B. Marshall, Lisa S. Kilpela

**Affiliations:** 1 ReACH Center, UT Health San Antonio, San Antonio, TX 78229, USA; 2 Barshop Institute for Longevity and Aging Studies, UT Health San Antonio, San Antonio, TX 78229, USA; 3 Division of Geriatrics, Gerontology and Palliative Medicine, Department of Medicine, UT Health San Antonio, San Antonio, TX 78229, USA; 4 Geriatrics Research, Education, and Clinical Center, South Texas VA Healthcare System, San Antonio, TX 78229, USA

**Keywords:** binge eating disorder, over nutrition, cardiometabolic health, physical functioning, body composition, post-menopausal

## Abstract

Binge eating disorder (BED), a form of overnutrition, may impact healthy aging for postmenopausal women. In community samples, 12–26% of older women (ages 60+) engage in binge eating. In younger adults, BED is comorbid with physical and psychological morbidities. However, little is known regarding the clinical phenotype, including medical and psychiatric comorbidities, of BED in postmenopausal women. This pilot study sought to identify psychosomatic, cardiometabolic, body composition, and physical function characteristics of postmenopausal, older adult (age ≥60 years) women with BED. Participants (*N* = 21, ages 60–75) completed a battery of physical assessments and surveys assessing psychosomatic health. Overall, 62% of women reported BE onset during peri- or post-menopause. Rates of comorbid depression, anxiety, sleep problems, and a history of severe menopausal symptoms were high. Cardiometabolic health was poor, and 42.9% met the criteria for metabolic syndrome. Additionally, 71.4% met the BMI criteria for obesity, and 40% of this sample met the criteria for sarcopenic obesity. Almost half of the sample presented with at least one mobility limitation; 85.7% had poor endurance. Evidence suggests that BED is highly comorbid with other chronic health conditions and may complicate treatment of these conditions, warranting further investigation and increased attention from healthcare providers serving postmenopausal women.

## 1. Introduction

The older adult population is growing at a rapid rate; by 2030 an estimated 20% of the U.S. population will be 65 years or older [1]. Notably, women continue to outlive men, yet they experience higher rates of morbidity later in life [2,3]. Additionally, women’s bodies undergo a host of changes as they enter and progress through midlife and late-life that may affect health and wellbeing. For instance, changes associated with the menopausal transition (e.g., metabolic, body composition, sleep, and lifestyle changes) comprise elevated risks for various cardiometabolic and musculoskeletal conditions [4,5]. Perimenopause is also associated with an increased risk for depression and other psychiatric and behavioral health disorders [6,7]. Though perimenopause is commonly studied as a window of vulnerability for certain psychiatric disorders, research suggests that postmenopausal women may also experience vulnerabilities to certain behavioral health disorders, which can impact healthy aging [8,9].

Importantly, nutrition pathology, including both under- and overnutrition, can negatively affect healthy aging. One form of overnutrition, binge eating (BE; defined as discrete episodes of consuming an abnormally large amount of food in one sitting while simultaneously feeling out of control), has emerged as a common form of eating pathology among older adult women. Although early research among older adults found prevalence rates of BE approximating 3.5–6% [10,11,12], recent estimates of BE behaviors among older women (aged 60+) in community samples fall between 12–26% [13,14]. Additionally, 19% of older women reported loss-of-control eating [13], regardless of food amount. Importantly, loss of control is more predictive of poorer outcomes than amount of food [15]. Ranges in prevalence rates could represent methodological variances, differences in operational definitions of active BE, or a birth cohort effect. Despite recent evidence of high prevalence rates in this population, few studies have described the experience of postmenopausal, older women with BE, especially geriatrics-relevant health correlates and comorbidities.

Of note, BE is linked with numerous medical problems in the general population. In younger adults, BE is associated with higher body mass index (BMI) and poorer inflammatory and metabolic profiles as compared to non-BE obese counterparts [16]. A disorder characterized by weekly BE (Binge Eating Disorder; BED) is associated with myriad medical morbidities, such as metabolic syndrome, sleep disturbances, cardiovascular issues, somatic symptoms, and disability [17,18]. Among older populations, BE may confer additional risk for multimorbidity, thus impacting healthy aging and quality of life for older adult women. However, older adults have been omitted from BED research [19,20], leaving the clinical significance and profile of BED in this population relatively unknown.

In the limited available data, BE in midlife and in perimenopausal women has garnered slightly more attention than BE in postmenopausal women. Research demonstrates mixed evidence regarding whether there is an increased risk for BE in midlife women that is directly related to menopause and associated hormonal changes [21]. However, overall disordered eating has been strongly associated with depression and with less physical activity among women aged 46–76 [22]. Additionally, among older women (*N* = 20; aged 65–77) with current BED, depression, anxiety, and elevated BMI were highly comorbid; 25% self-reported hypertension or hypercholesterolemia, while no participants self-disclosed a diabetes diagnosis [23]. In a general sample of postmenopausal women (*N* = 227; aged = 60–94), retrospective menopausal symptom severity was significantly associated with BE severity [24]. The medical history data in these samples are limited by the exclusive use of self-reported past medical conditions (versus the use of electronic medical record data to verify medical history).Thus, much remains to be learned about the clinical phenotype and disease burden of BE in later life.

The goal of the current pilot study was to describe the psychosomatic, cardiometabolic, body composition, and physical function characteristics of postmenopausal, older adult (age ≥60 years) women with current BED as preliminary data for the clinical presentation of BE in later life. According to the Diagnostic and Statistical Manual for Mental Disorders (DSM-5), BE ≥1 per week over ≥3 months represents the frequency and duration minimums for a diagnosis of BED [25]. Additionally, we collected data on depressive symptoms, anxiety, and sleep problems, as all are highly comorbid with BED [26,27]. Lastly, we collected data on retrospective menopausal symptoms. We benchmarked our results using age- and gender-matched data from the general population. Data describing the clinical presentation of postmenopausal women with BED in later life, including considerations for multimorbidity dimensions, will help us to best identify both treatment needs and approaches for this population. Ultimately, the effective treatment of BED among older women may be one avenue to improve quality of life and increase longevity in postmenopausal women.

## 2. Method

### 2.1. Procedure

This study received ethics approval from the Institutional Review Board, and all participants gave written informed consent. Participants were recruited through online postings, flyers displayed in local senior centers and outpatient clinics, on institutional websites, and through a third-party patient recruitment service (TrialFacts). Advertisements (with binge definition provided) sought women aged 60+ who had experienced a binge episode in the past month. Interested individuals underwent a phone screen to determine eligibility. Individuals were eligible to participate if they were a female aged 60+, endorsed objective binge episodes ≥1/week over the past ≥3 months, had no medication changes in the past three months, were community dwelling, and were not currently receiving treatment for an eating pathology.

Evaluation during phone interviews included the binge episode diagnostic items from the Eating Disorders Examination (EDE-5) [28] and were conducted by intensively trained research assistants supervised by a clinical psychologist (LSK) who reviewed all interviews. All diagnoses of BED were confirmed by the licensed clinical psychologist and eating disorders expert (LSK). Interviews confirmed the presence of objective binge episodes (i.e., discrete episodes of consuming an abnormally large amount of food in one sitting while feeling out of control at that time), the frequency of binge episodes, and the duration of BE illness (based on the retrospective reporting of BE onset). At the time of each participant’s consent visit, two final eligibility assessments were completed before formally enrolling them into the study—the Mini Mental State Examination, and an assessment for acute psychosis/suicidality.

Following consent/enrollment, participants completed a survey and one in-person visit that included a comprehensive battery of physical health assessments. The survey, completed digitally, assessed psychiatric and physical health history, as well as current mental health. During the physical health assessment visit, participants underwent cardiometabolic, body composition, and physical functioning assessments. Participants were compensated with a $75 university-issued gift card at the completion of the study.

### 2.2. In-Lab Measures

#### 2.2.1. Cardiometabolic

Blood pressure was measured for each participant, as well as fasting HbA1c, a lipids panel, and a comprehensive metabolic panel (CMP), and Vitamin D.

#### 2.2.2. Body Composition

We collected measurements of height/weight (to calculate BMI), and waist-to-hip ratios. Measurements were collected in triplicate and averaged. To assess body composition in terms of fat and fat-free mass, we used Dual Energy X-ray Absorptiometry (DXA). We also used DXA to examine appendicular lean mass to help identify any indications of sarcopenic obesity, which refers to reduced muscle mass in the context of high adiposity [29]. According to the Foundation for the National Institutes of Health, an individual meets the criteria for sarcopenic obesity if they have an appendicular lean mass (ALM)/BMI of <0.512 and a BMI defined as obese (≥30 kg/m^2^) [30]. While sarcopenia (i.e., loss of muscle mass and strength or function that occurs with aging) is often considered in the context of low weight and frailty, sarcopenia and obesity share similar underlying mechanisms, such as hormones, lifestyle behaviors, and immunological factors [31] Thus, shared mechanisms of obesity and sarcopenia may act synergistically to increase the risk for and/or have a greater effect on cardiovascular disease and metabolic disorders [31]. Sarcopenic obesity affects 18% of older women nationally [30], and it increases the risk of physical dysfunction, disability, falls, and mortality [32,33,34].

#### 2.2.3. Physical Function

To assess physical function, we administered the Short Physical Performance Battery [35]. This assessment was designed for older adults to measure balance, gait speed, and lower extremity strength. It is sensitive to changes in physical function (0.5–1.0 point change is clinically significant) and has high reliability (ICC = 0.88–0.92). A score lower than 10 suggests mobility limitations and predicts all-cause mortality. Grip strength was assessed using a handheld dynamometer. For each participant, their dominant hand was tested, or the hand most comfortable for the participant in the event of past hand or wrist injury/surgery. Participants completed this assessment three times; the average of the three trials was their final grip strength score.

Endurance was measured using the 6-min walk test [36]. This assessment was designed for older adults, and measures how far an individual can walk in six minutes. Due to limited space in the study facility, we used the recommendation of a 40-foot indoor track instead of the standard 30-m track for this assessment. Participants were allowed to use assistive devices if needed, and this was noted by the research team. Not only are these assessments commonly used with older adults so as to limit possible injury, but we implemented additional precautions to ensure the physical safety of all participants.

### 2.3. Self-Report Measures

Regarding demographics, participants self-reported age, race/ethnicity, education, relationship status, household income, self-reported medical history, current prescription medications, and any current or past hormone replacement therapy.

To measure depression, we used the 10-item Center for Epidemiologic Studies–Depression Scale (CES-D) [37]. Items were rated on a 4-point scale and are summed for the total score, with higher scores indicating greater depressive symptoms. A score of 10 or greater indicates clinical depression (current sample α = 0.88).

The Geriatric Anxiety Inventory Short Form (GAI-SF) [38] was used to detect anxiety symptoms. This scale contains seven self-report items to measure anxiety symptoms over the previous week. The scores are summed, with higher scores indicating greater anxiety symptoms. A score of 2 or greater indicates a probable anxiety disorder. The internal consistency for this sample was good (α = 0.85).

To assess sleep quality, we used the Pittsburgh Sleep Quality Index (PSQI) [39]. This measure contains 19 self-rated questions, each with a range of 0–21. The questions are combined to form seven component scores that are summed to yield a global PSQI score. Global scores range from 0–21, and higher scores indicate poorer sleep quality. A score of >5 indicates clinical-level sleep problems. The internal consistency in the current sample was good (α = 0.80).

We used the Menopause Rating Scale (MRS) [40] to assess retrospective menopause symptoms (α = 0.86). The MRS has three subscales: psychological, somato-vegetative, and urogenital symptoms. The scores are summed to obtain a total score, with higher scores indicating more severe symptoms. Total scores are categorized by severity: none, or little (0–4), mild (5–8), moderate (9–16), and severe (17+) [41].

## 3. Results

### 3.1. Participants

Participants included 21 postmenopausal women, aged 60–75. Regarding race/ethnicity, 47.61% were non-Hispanic White race, 4.76% were Black, 14.29% identified as mixed race, and 33.33% were of Hispanic ethnicity. Over half of this sample (61.9%) had a Bachelor’s degree or higher. Additionally, 38.1% were married and 42.86% were retired (Table 1). The average age of onset for menopause in this sample was 46.57 ± 6.68; 28.6% reported having a medical condition or undergoing a procedure that induced menopause. Additionally, 23.8% reported currently or previously taking Hormone Replacement Therapy. Of note, only 38.1% of this sample reported BE onset before menopause, indicating that the majority developed BE during peri- or post-menopause. See Table 2 for descriptive data and effect sizes for all comorbidity indicators.

### 3.2. Psychosomatic Comorbidities

Regarding psychological health, 61.9% of this sample met the clinical cutoff for depression, 57.1% for an anxiety disorder, and 81% for clinical sleep problems. Regarding the severity of symptoms during menopause, the majority (76.2%) of participants reported either moderate (28.6%) or severe symptoms (47.6%). Two sample t-tests were conducted using the means and standard deviations of all psychosomatic comorbidities in this sample compared to nonclinical, community dwelling older adults. The current sample had significantly higher average scores on symptoms of depression [*t*(969) = −2.0, *p* = 0.046, 95%CI (−7.23, −0.07)], anxiety [*t*(78) = −7.74, *p* < 0.001, 95%CI (−2.84, −1.68)], sleep problems [*t*(263) = −3.76, *p* = 0.002, 95%CI (−5.18, −1.62], and menopause symptoms [*t*(1395) = −4.32, *p* < 0.001, 95%CI (−10.52, −3.96)] compared to community samples [41,42,43,44]; higher averages indicate greater pathology. The effect size for depressive symptoms was medium; all other effect sizes were either medium-to-large or large in magnitude (see Table 2) [41,42,43,44].

### 3.3. Cardiometabolic

According to in-lab assessments, 47.61% of participants had high fasting blood glucose levels (>99 mg/dL), 42.86% had high cholesterol (>199 mg/dL), 76% had high LDL cholesterol (>99 mg/dL), and 33.33% had low Vitamin D (<30 ng/mL). Only one participant (4.8%) had low total protein levels (<6.0 mg/dL), and two participants (9.5%) had low HDL cholesterol (<39 mg/dL). Additionally, 52.38% had pre-diabetes and 9.5% (*n* = 2) of participants had diabetes; according to the CDC, 48.8% of adults aged 65+ in the general population have prediabetes and 24.4% have diabetes [45]. Of note, nine women (43%) in the current sample reported a history of diabetes or pre-diabetes; four other women denied any pre/diabetes history but had HbA1c values in the pre-diabetic range based on samples collected in this study. Thus, HbA1c levels for 62% of the participants were in the pre-diabetic or diabetic range, even with medications.

Two sample t-tests were conducted using the means and standard deviations of cardiometabolic outcomes in this sample compared to nonclinical, community dwelling older adults. Both glucose [*t*(1709) = −7.97, *p* < 0.001, 95%CI (−22.98, −13.90)] and HbA1c [*t*(1709) = −7.01, *p* < 0.001, 95%CI (−0.83, −0.47)] levels in the current sample were significantly higher overall than in older adults from the Health, Aging, and Composition study [46]. The effect sizes for glucose and HbA1c were medium-to-large in magnitude (Table 2) [46]. Notably, there are limited data on cholesterol norms in community dwelling, nonclinical samples of older adults. Therefore, cholesterol levels from this study were benchmarked to treatment-seeking older adults [47]. LDL cholesterol levels were significantly lower [*t*(535) = 2.02, *p* = 0.04, 95%CI (.44, 32.94)] and HDL cholesterol levels were significantly higher [*t*(535) = −3.10, *p* = 0.002, 95%CI (−15.92, −3.56)] in the current sample compared to treatment seeking older women; cholesterol levels were not significantly different between the two groups [47]. The effect sizes were medium in magnitude for total cholesterol and LDL cholesterol levels, and medium-to-large in magnitude for HDL cholesterol levels (Table 2).

Regarding blood pressure, nine participants (43%) reported a history of hypertension; 8/9 reported current antihypertensive use and did not have hypertension in the lab. Only one participant had standard hypertension according to in-lab assessments, and she denied medication use. Of the total sample, 33.33% had isolated systolic hypertension and 28.37% had elevated blood pressure (120–129). Overall, systolic [*t*(177) = 4.18, *p* < 0.001, 95%CI (10.17, 29.37)] and diastolic [*t*(177) = 6.72, *p* < 0.001, 95%CI (10.08, 18.46)] blood pressure in this sample were significantly lower compared to age-matched norms [48], and the effect size was large (Table 2). Thus, hypertension in the context of BED may be managed well with the use of medication.

Furthermore, we investigated whether participants met the criteria for metabolic syndrome (MetS). Participants were categorized as having MetS if they met at least three of the following criteria: a waist circumference of >35 inches, >130 systolic BP and >85 diastolic BP, fasting blood glucose ≥ 100 mg/dL, HDL cholesterol <50 mg/dL, or triglycerides ≥150 mg/dL [49]. Based on this criterion, 42.9% (*n* = 9) of women had MetS; national data suggest approximately 50% of women aged 60+ have MetS [49,50].

### 3.4. Body Composition

Regarding body composition, 19% of this sample had a BMI classified as overweight (25.0 ≤ BMI <30), and 71.4% had a BMI classified as obese (BMI ≥30), compared to 44.2% of women 60+ in the general population being classified as obese [45]. Additionally, 85.7% had a waist-to-hip ratio of 0.86 or above, indicative of all-cause mortality [51]. Two sample t-tests were conducted using the means and standard deviations of body composition metrics in this sample compared to nonclinical, community dwelling older adults [52,53]. When compared to age-and gender-matched norms, BMI [*t*(1356) = −6.23, *p* < 0.001, 95%CI (−9.84, −5.12)], total body fat percentage [*t*(1355) = −7.20, *p* < 0.001, 95%CI (−12.34, −7.06)], and waist-to-hip ratio [t(1049) = −53.49, *p* < 0.001, 95%CI (−0.082, −0.077] were significantly greater in the current sample [52,53]. Effect sizes were large (Table 2) [52,53]. Based on the Foundation for the National Institutes of Health recommended cutoff of <0.512 for ALM/BMI, 40% of the sample met the criteria for sarcopenic obesity [30].

### 3.5. Physical Function

Regarding physical function, 47.62% of women had a score <10 on the SPPB, indicating one or more mobility limitations and predictive of all-cause mortality [35]. Additionally, 18.05% had a grip strength 1SD below the age- and gender-matched norm [54]. Two sample t-tests were conducted using the means and standard deviations in this sample compared to nonclinical, community dwelling older adults. There were no significant differences in physical functioning according to the SPPB or differences in grip strength between the current sample and community norms [55]. However, 85.7% of participants were 1SD below the age and gender matched norm for the distance walked during the 6-min walk [56], and the current sample had significantly lower scores on the 6-min walk [*t*(41) = 7.39, *p* < 0.001, 95% CI (132.26, 231.82)] compared to an age and gender matched community sample [56]. The effect size was large (Table 2). Of note, one participant used a cane for all physical function assessments and one participant (who had arthritis and multiple sclerosis) used her cane only for the 6-min walk. An additional participant brought her walking aid but did not use it for any assessments.

**Table 2:** Descriptive statistics and effect sizes for all outcomes in the current sample versus norm-based, community samples.

Assessment	*M* (SD)	Min–Max	Community Sample *M* (SD)	Cohen’s *d* ^§^
*Cardiometabolic*				
Blood Pressure				
Systolic	133.93 (18.74)	100.33–173.67	153.2 (20) ^a^	−0.99 ^a^
Diastolic	67.33 (9.42)	52.67–93.67	81.6 (9.1) ^a^	−1.54 ^a^
Blood Labs				
Glucose (mg/dL)	111.24 (43.27)	82–273	92.8 (9.5) ^b^	0.59 ^b^
Cholesterol(mg/dL) ^¥^	193.52 (36.51)	95–278	211.2 (42.6) ^c^	−0.45 ^c^
LDL Cholesterol (mg/dL) ^¥^	112.71 (27.21)	36–171	129.4 (37.5) ^c^	−0.51 ^c^
HDL Cholesterol (mg/dL)	62.24 (17.27)	34–101	52.5 (14.0) ^c^	0.62 ^c^
HbA1c (%)	5.95 (1.32)	4.70–11.20	5.3 (0.4) ^b^	0.67 ^b^
Vitamin D (ng/mL)	35.06 (11.13)	20.50–64.30	-	-
Protein, total (g/dL)	6.82 (0.32)	5.9–7.3	-	-
*Body Composition*				
BMI	35.08 (8.64)	21.20–56.60	27.6 (5.4) ^d^	1.04 ^d^
Waist-to-Hip Ratio	0.9 (0.047)	0.82–1.01	0.82 (0.002) ^e^	2.4 ^e^
DXA (*n* = 20)				
Total Body Fat %	47.0 (4.5)	38.9–54.3	37.3 (6.0) ^d^	1.83 ^d^
AM Fat Percentile	84.75 (17.43)	45–99	-	-
ALM/BMI	0.54 (0.08)	0.46–0.82	-	-
*Physical Functioning*				
SPPB Total	9.43 (2.25)	5–12	8.3 (2.7) ^f^	0.45 ^f^
Six Minute Walk (meters)	355.96 (67.04)	181.66–451.40	538 (92) ^g^	2.26 ^g^
Grip Strength (kg)	24.91 (6.40)	24.57–40.37	25.3 (4.8) ^h^	0.07 ^h^
*Psychosomatic Comorbidities*				
Depression	11.95 (6.94)	1.0–24.0	8.3 (8.3) ^i^	0.48 ^i^
Anxiety	2.43 (2.01)	0.0–5.0	0.17 (0.62) ^j^	1.52 ^j^
Sleep Problems	10.0 (4.77)	2.0–17.0	6.6 (3.9) ^k^	0.88 ^k^
Menopause Symptoms	16.34 (8.55)	3.0–33.0	9.1 (7.6) ^l^	0.90 ^l^

Notes: ^§^ Current sample means and standard deviations compared to norms in community dwelling older adults; ^a^ Budge et al., 2002 [47]; ^¥^ Comparison group comprised treatment seeking older adults; ^b^ Lipska et al., 2013 [45]; ^c^ Cabrera et al., 2007 [46]; ^d^ Newman et al., 2003 [52]; ^e^ Dobbelsteyn et al., 2001 [53]; ^f^ Perera et al., 2006 [55]; ^g^ Steffen et al., 2002 [56]; ^h^ Desrosiers et al., 1995 [54]; ^i^ Tomita & Burns, 2013 [42]; ^j^ Johnco et al., 2015 [43]; ^k^ Beaudreau et al., 2012 [44]; ^l^ Heinemann et al., 2004 ^41^; Cohen’s *d* effect size interpretation: small = 0.2, medium = 0.5, large = 0.8.

## 4. Discussion

This pilot study sought to identify psychosomatic cardiometabolic, body composition, and physical function and characteristics of postmenopausal, older adult (age ≥ 60 years) women with current BED. Overall, postmenopausal women with BED presented with not only multiple psychological comorbidities, but medical morbidities as well. Most (61.9%) women reported BE onset during peri- or post-menopause. Rates of comorbid depression, anxiety, sleep problems, and a history of severe menopausal symptoms were high in this sample. Additionally, cardiometabolic health was poor, as many women had high glucose and cholesterol levels, and poorly controlled pre-diabetes or diabetes. Almost half of the sample also met the criteria for metabolic syndrome [49]; this is based on in-lab assessments and does not account for the effects of medication on meeting criteria. Furthermore, a significant majority met the BMI criteria (≥30 kg/m^2^) [57] for obesity in this sample, and almost half of the sample met the criteria for sarcopenic obesity. Finally, almost half of the sample presented with at least one mobility limitation; the vast majority of women in this sample had poor endurance, scoring more than 1SD below age- and gender-matched norms [56].

While this study is descriptive in nature, identifying this clinical phenotype of older women with BED has several implications. For instance, it is imperative to increase awareness among healthcare professionals, especially those who see older adult patients, regarding the screening and diagnosis of BED in older populations. The awareness of BED among older populations is important for two main reasons. First, BED is evidently highly comorbid with other chronic health conditions that are common among postmenopausal women. Thus, the presence of comorbid BED will likely complicate the management of other chronic conditions if left unaddressed within an overall treatment regimen. Whether comorbid BED exacerbates other chronic health conditions among postmenopausal women over time remains unknown, and further data are needed to determine the chronological health burden of BED in older populations.

Additionally, behavioral health treatments that target obesity and diabetes are primarily aimed at weight loss through obtaining and maintaining a state of energy deprivation [58]. However, BED treatments aim to regulate intake (e.g., timing of intake, amount, type) and output to achieve and maintain energy balance [59]. States of deprivation often precede BE episodes [60]; thus, weight loss programs can elicit new onset BE over time [61]. These data suggest that behavioral treatments used to target obesity or diabetes that do not consider BED are likely to be less effective and potentially even harmful when BE episodes are present. Thus, it is important that medical providers screen for BED in older adult women and consider BED when recommending behavioral health treatments. In these cases, interventions targeting BE episodes, as well as consideration for psychiatric comorbidities (e.g., depression, sleep disturbances), are needed.

Furthermore, though evidence-based treatments (EBTs) for BED exist, there are no guidelines for treating postmenopausal women, as they have been consistently excluded from BED treatment research [23,62]. In 58 eating disorder randomized control trials, only three even allowed participants age ≥ 65 years; none were designed for older adults [63]. Current EBTs, such as cognitive behavioral therapy, do not address medical morbidities that may accompany BED in older women, nor do they account for aging-related changes in cognition or psychosocial factors [64]. These treatments commonly target body dissatisfaction and similar mechanisms during treatment, which do seem to be present in eating disorders through midlife [65]. However, given the medical morbidities in the current sample, we likely need a multifaceted treatment approach that not only helps older women stop BE, but increases physical health and mobility as well. Thus, more research dedicated to identifying how best to treat BED in older women that results in the cessation of BE, as well as in improved physical and mental health, is warranted.

It is important to note that this study has several limitations. While the data presented are pilot data, the sample size is small. We do not have data on the current use of Vitamin D or other vitamins/supplements; future research should collect data on supplement use as well as prescription medications. Additionally, the cross-sectional design limits the ability to draw conclusions about the direct impact BED may have on the physical health of older women, and vice versa. Longitudinal research examining the prospective relations of cardiometabolic health and physical function in postmenopausal women with BED is needed.

Furthermore, there is no control group in the current study, so results from this study were compared to age- and gender-matched population norms when available. Using norm-based comparisons comes with both benefits and costs. One benefit in particular is the ability to benchmark (including the ability to age- and gender-match) clinical samples to large, non-clinical or community samples, which may have smaller confidence intervals or variance than smaller, comparison samples. Smaller, independently collected control samples may be subject to sampling or other biases at local recruitment sites (i.e., further contributing to the replication crisis in psychological science [66]).

Alternatively, norm-based data often come in large sample sizes which are accompanied by several limitations, such as the overdetection of small effects, the detection of rare effects, and the overreliance on *p*-values or statistical significance [67]. Therefore, if there are biases inherent in the large, norm-based sample that is consistently being used as a benchmark to clinical populations, it can result in systematic errors in estimating effect sizes. Recommendations by Lin and colleagues [67] to overcome these weaknesses include the approximation of effect sizes and confidence intervals. It is worthy of note that we included these additional statistics when comparing our data to the norm data. However, given these potential limitations of large samples sizes, the results of the comparison of our sample to norms should be interpreted with caution.

## 5. Conclusions

Overall, these pilot data suggest that further research into the physical consequences of engaging in BE for postmenopausal women is warranted. Additionally, it is imperative that future research examine current BED treatments and how we can address multimorbidity, not just psychological comorbidities, in treatments to increase the quality of life and health span of postmenopausal, older adult women.

## Figures and Tables

**Table 1:** Demographics and self-reported medical history (*N* = 21).

Demographics	*M* (SD) or *N* (%)
Age	66.0 (4.59)
BMI	35.08 (8.64)
Race/Ethnicity	
Non-Hispanic White	10 (47.62%)
Hispanic/Latino	7 (33.33%)
African American/Black	1 (9.5%)
Mixed Race	3 (14.29%)
Marital Status	
Married or living with partner	8 (38.1%)
Single or divorced/separated	10 (47.7%)
Widowed	2 (9.5%)
Education	
Graduated high school or GED	2 (9.5%)
Some college	5 (23.8%)
Graduated 2-year college	1 (4.8%)
Graduated 4 year college	5 (23.8%)
Part graduate school	4 (19%)
Graduate school degree	4 (19%)
Income	
10,000–35,000	4 (19%)
35,000–50,000	3 (14.3%)
50,000–75,000	4 (19%)
75,000–100,000	4 (19%)
100,000+	3 (14.3%)
High blood pressure *	9 (42.86%)
Heart problems *	2 (9.5%)
Breathing or lung problems *	7 (33.33%)
Pre-diabetes	11 (52.4%)
Diabetes	2 (9.5%)

Note. * Self-reported current and/or past medical history.

## Data Availability

The data presented in this study are available on request from the corresponding author. The data are not publicly available due to the pilot nature of this study affecting the ability to truly anonymize clinical profiles among participants.
